# Inflammation and enhanced nociceptive responses to bladder distension produced by intravesical zymosan in the rat

**DOI:** 10.1186/1471-2490-6-2

**Published:** 2006-02-09

**Authors:** Alan Randich, Tyler Uzzell, Ronda Cannon, Timothy J Ness

**Affiliations:** 1 Department of Psychology, University of Alabama at Birmingham, Birmingham, Al, 35294, USA; 2 Department of Anesthesiology, University of Alabama at Birmingham, Birmingham, Al, 35294, USA

## Abstract

**Background:**

Mycotic infections of the bladder produce pain and inflammatory changes. The present study examined the inflammatory and nociceptive effects of the yeast cell wall component, zymosan, when admininstered into the urinary bladder in order to characterize this form of bladder sensitization.

**Methods:**

Parametric analyses of the time-course (0–48 hr) and concentration (0–2% solutions) variables associated with intravesical zymosan-induced bladder inflammation were performed in female rats. Plasma extravasation of Evan's Blue dye was used as a measure of tissue inflammation. Cardiovascular and visceromotor responses to urinary bladder distension were used as measures of nociception.

**Results:**

Zymosan-induced bladder inflammation, as indexed by plasma extravasation of Evan's Blue, was significantly greater in rats treated with either 1 or 2% solutions as compared to either 0.1 or 0.5% zymosan solutions. In time-course studies (1 – 48 hr post-treatment), 1% zymosan-induced inflammation progressively increased with time following administration, was greatest at 24 hr and began to normalize by 48 hr. In the studies of inflammation-induced changes in nociception, arterial blood pressure (ABP) and visceromotor responses to graded distension of the urinary bladder were significantly increased relative to controls 24 hr after zymosan administration.

**Conclusion:**

These studies provide important time-course and solution concentration parameters for studies of zymosan-induced inflammation of the bladder and suggest utility of this model for the study of bladder-related pain.

## Background

Our knowledge of inflammation-induced changes in bladder tissue that result in bladder-related pain is limited. Bacterial, mycotic and viral infections of the urinary tract are all potential etiologies of bladder inflammation [1]. Our understanding of the neural substrates that are altered by infectious processes is virtually nonexistent. Recent studies have demonstrated that the injection of the substance lipopolysaccharide (LPS), derived from the cell wall of *E. Coli *bacteria, activates the innate immune mechanisms (Toll-like receptor 4-related) that are normally activated by infection [2]. Mycotic infections, such as those produced by *Candida Albicans*, activate similar, but distinctly different innate immune mechanisms [3,4]. Zymosan is a mycotic equivalent to bacterial LPS in that it is yeast cell wall derivative which activates the Toll-like receptor 2 (TLR2) and the alternate complement pathway [4]. Zymosan can be viewed as a "natural" inflammogen which produces a receptor-mediated reactive cellular process as opposed to chemical irritants which result in frank, extensive tissue damage that leads to inflammation. Zymosan has been extensively characterized in behavioral, primary afferent, and dorsal horn neuron studies of cutaneous inflammation [e.g., [5,6]]. A robust colitis also is produced by intracolonic administration of zymosan [7], but no one has reported on the use of zymosan in other visceral structures.

Only a few validated animal models of visceral nociception exist (for review, see [8]). Those that have been validated typically have used observations of animal posturing as well as reflex increases in arterial blood pressure (ABP) and electromyographic (EMG) activity of the abdominal muscles (visceromotor reflex) as response measures of visceral nociception. The presence of inflammation has been demonstrated to be a potent enhancer of responses to visceral stimuli. In particular, inflammation of the colorectal mucosa by the intracolonic application of turpentine [9,10], acetic acid [11,12], trinitrobenzene sulfonic acid [13] as well as zymosan [7] has been demonstrated to magnify reflex ABP and EMG responses to distension of the colon (CRD). In humans, inflammation also frequently leads to an enhancement of visceral pain sensations and so animal models demonstrating an enhancement of responses to a visceral stimulus produced by inflammation support the use of the models as behavioral indices of nociception.

Recently, we [14,15] proposed using similar response measures evoked by urinary bladder distension (UBD) as an animal model of bladder pain. In our initial studies, we found these measures to be reliable and valid indices of bladder nociception in the rat with responses inhibited by analgesic manipulations. In the present studies, we sought to determine whether these response measures were affected by inflammation of the urinary bladder using zymosan as an inflammogen by determining the optimal concentration and time-course parameters for producing zymosan-related inflammation of the bladder as measured by Evan's Blue-related plasma extravasation and then correlating these inflammatory changes with nociceptive responses evoked by UBD of the inflamed bladder. Plasma extravasation of Evan's Blue dye has been validated as a measure of local inflammation in numerous previous studies [16-18].

## Methods

### General

Adult, female Sprague Dawley rats were obtained from Harlan Laboratories (Prattville, AL) and housed in group cages. Food and water were provided on an *ad libitum *basis. These studies were approved by the University of Alabama at Birmingham Institutional Animal Care and Use Committee.

### Experiment 1 – Time-course of inflammatory responses

This experiment determined the time-course of inflammation produced by the intravesical administration of zymosan into the urinary bladder. Inflammation was measured as a function of the amount of Evan's Blue plasma extravasation into the bladder using a slight variation on previously published methodology [10,16-18]. Separate groups of rats (N = 5 for all groups) received either no treatment (naive group), or received intravesical administration of either saline (0.5 ml) or a 1% zymosan solution (0.5 ml). Rats receiving treatments were anesthetized with halothane (1–5%) by mask to provide immobility for the placement of a 22-gauge angiocatheter into the urinary bladder via the urethra without trauma. Solutions were then administered and left in place for 30 minutes. Bladders were then drained and rats recovered from anesthesia. To prevent the introduction of infection into the bladders a povidone surface preparation was performed prior to catheterization and each treated rat received 50–100 mg/kg of ampicillin (s.c.). Groups of rats receiving zymosan were tested 1, 4, 12, 24, or 48 hr after their treatment. The saline control group rats were tested 24 hr after their treatment. Saline administration was used to control for all aspects of the procedure excluding inflammation, e.g., placement of the transurethral catheter and bladder distension. At the time of testing, all rats were reanesthetized and administered Evan's Blue (50 mg/kg i.v. via external jugular) which was allowed to circulate for 15 min. Animals were euthanized with pentobarbital (100 mg/kg i.v.) and perfused intracardially with 500 ml of normal saline. Whole bladders were removed and placed in 10 ml of dimethylsulfoxide for subsequent analysis. The amount of Evan's Blue extracted from the bladder tissue was determined spectrophotometrically (620 nm) and data were expressed in μg/g of bladder weight.

### Experiment 2 – Effect of Zymosan concentration

This experiment determined the effect of zymosan concentration on the degree of plasma extravasation of Evan's Blue in the bladder. The same general procedures were used as described in Experiment 1 except that separate groups of rats (N = 5 per group) received intravesical administration of differing concentrations of zymosan (0.1, 0.5, 1.0, and 2.0%) in normal saline and the Evan's Blue determination was performed 24 hr later. The 1% zymosan data were those derived from Experiment 1.

### Experiment 3 – Time-course of effects on nociceptive reflexes

This experiment determined the time-course of the effects of intravesical administration of zymosan on abdominal EMG and systemic ABP responses to UBD. Separate groups of rats (N = 6–21/group) were treated similarly to those described in Experiment 1: rats received either no treatment (naive), or intravesical administration of either saline (0.5 ml) or a 1% zymosan solution (0.5 ml). Zymosan-treated rats were tested at 4, 12, 24, or 48 hr after their treatment and the saline control group was tested at 24 hr. Methodology is the same as that previously published [15]. Briefly, at the time of testing, each rat was re-anesthetized with halothane and instrumented for cardiovascular and EMG measures. Carotid arterial and tracheal cannulae were inserted for recording of ABP and artificial respiration, respectively under deep halothane anesthesia (2–5%). The right vagus nerve is sometimes injured during carotid manipulation and so to provide consistency between preparations was formally transected. The left abdominal skin was incised and silver wires were sutured to the external oblique abdominal musculature allowing for differential amplification of EMG activity. A 22-gauge angiocatheter was placed into the urinary bladder via the urethra and held in place by a tight ligature around the distal urethral orifice.

Following completion of the surgical preparation, the halothane anesthesia was reduced until flexion reflex responses could be evoked by pinch of the foot but without spontaneous escape behaviors (typically 0.5–0.7% halothane). The rats were artificially ventilated and not restrained in any fashion. Body temperature was maintained using a heating pad and overhead radiant lights. All data were saved on computer using MacIntosh-based Spike-2 software and associated hardware (Micro 1401; CED, Cambridge, UK) and a similar MacLab system (Stoelting, Inc).

All rats received multiple distensions of the urinary bladder via the intravesical catheter. An in-line, pneumatically-linked, low volume pressure transducer was used to monitor intravesical pressure. Pressure within the bladder was controlled using a pressure control device [19]. Three 60 mmHg, 20 second UBDs (4 min intertrial interval) were administered followed by 20 second, constant-pressure air distensions of the urinary bladder at pressures of 10, 20, 30, 40, 50, and 60 mmHg, respectively.

ABP was measured continuously via the pulse pressure signal from the arterial cannula. In tests of UBD, an ABP response was defined as the peak change in mean ABP (?mmHg) during UBD as compared with the average response during a baseline period immediately prior to UBD. The EMG response was electronically rectified and normalized to baseline electronic "noise" levels (the lowest EMG activity level recorded from any particular rat.) These signal-to-noise ratios were defined as the EMG response to UBD and consisted of the following: (mean rectified EMG activity during UBD – mean rectified baseline EMG measured immediately prior to UBD) / (mean rectified baseline EMG noise level).

### Statistics

Data are presented as the mean ± S.E.M. unless otherwise stated. ABP and EMG responses to UBD are presented as change from baseline measures. One way ANOVAs were used for Evan's Blue data and repeated measures ANOVAs were used for ABP and EMG. Statistical significance was defined as p values ≤ 0.05.

## Results

### Experiment 1 – Time course of inflammatory response

**Figure 1:**
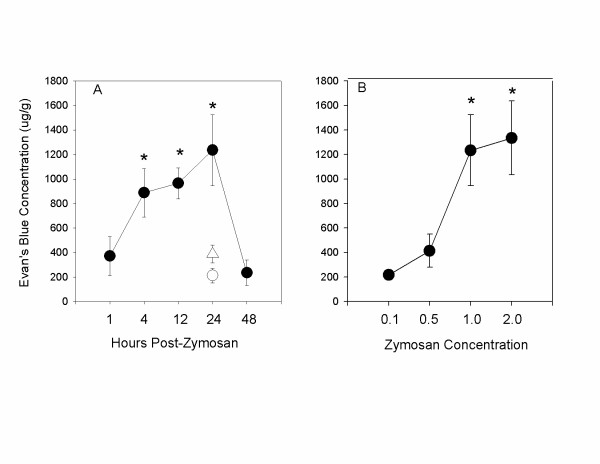
Indices of bladder inflammation using Evan's Blue extravasation as an experimental endpoint. Panel A presents group mean Evan's Blue concentration (μg/g) obtained 1, 4, 12, 24, or 48 hr after intravesical administration of 1% zymosan. Groups 4 hr 12 hr, and 24 hr did not differ from each other but were significantly greater than either groups 1 hr, 48 hr, naive control (open circle), and saline control (open triangle) as denoted by *. Panel B presents group mean Evan's Blue concentration obtained from rats receiving 0.1, 0.5, 1.0, or 2.0% intravesical zymosan administration 24 h previously. * denote significantly different from 0.1 and 0.5% groups. For all groups in A&B, N = 5. Error bars indicate SEM.

Panel A of Figure 1 shows that group mean plasma extravasation of Evan's Blue systematically increased from 1 to 24 hr after intravesical administration of 1% zymosan and then returned to low levels by 48 hr. A between-groups ANOVA revealed a significant effect of time and Fisher's post-hoc comparisons indicated that the 4, 12, and 24 hr zymosan groups were significantly greater than either the 1 hr or 48 hr zymosan groups (p < 0.05), but did not differ from one another. In addition, the 4, 12, and 24 hr zymosan group means were significantly greater than those of the non-pretreated (naive) and saline-treated control groups (p < 0.05).

### Experiment 2 – Effect of Zymosan concentration

Panel B of Figure 1 shows that the amount of plasma extravasation of Evan's Blue systematically increased as a function of the concentration of zymosan instilled into the bladder 24 hr prior to testing. An ANOVA revealed significant between-groups differences and Fisher post-hoc comparisons indicated that the 0.1% and 0.5% group means did not differ, but that they were significantly less than either the 1.0% and 2.0% groups whereas the latter two groups did not differ.

### Experiment 3 – Time-course of effects on nociceptive reflexes

**Figure 2:**
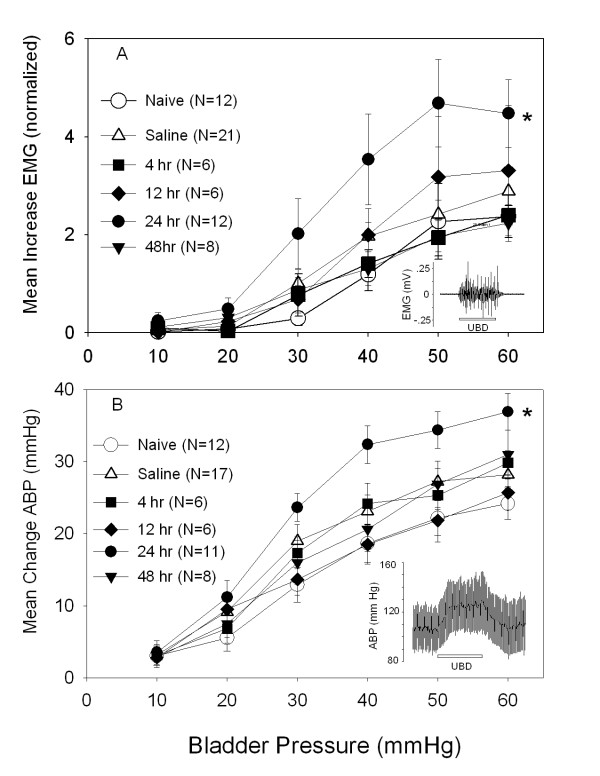
Enhancement of nociceptive responses to urinary bladder distension (UBD). Panel A presents group mean EMG as a function of UBD pressure – a typical EMG response to a 60 mm Hg UBD is given as an inset. The 24 hr zymosan-treated group differed significantly (p < 0.05) from the naive and saline control groups as denoted by *. Panel B presents group mean ABP as a function of UBD – a typical pressor response to a 60 mm Hg UBD is given as an inset. The 24 hr zymosan-treated group differed significantly from the naive and saline control groups as denoted by *. In A & B all other comparisons were not statistically significant. Note: the number of subjects given in B is less than those in A due to occasional technical difficulty with ABP measurements. Symbols are per inset legend (controls with open symbols; zymosan-treated with filled symbols). Error bars indicate SEM.

Panel A of Figure 2 presents the group mean EMG responses evoked by UBD in groups tested at various times after bladder instillation of a 1% zymosan solution. Only the 24 hr post-zymosan condition showed enhanced EMG responses compared to naive and saline control groups. An ANOVA indicated a significant overall group effect, F(5,59) = 3.16. The subsequent post-hoc comparisons revealed that the group mean EMGs of the naive and saline groups did not significantly differ, but that the means of these groups combined were significantly less than the group mean EMG of the 24 hr group. Similar comparisons of the naive and saline control data with all other zymosan time conditions failed to attain statistical significance.

Panel B of Figure 2 shows the group mean ABP responses to UBD in the same groups of rats described in Figure 2A. As was true of the EMG measures, only the 24 hr post-zymosan condition showed enhanced ABP responses to UBD with all other groups showing responses of comparable magnitude to both the naive and saline control groups. An ANOVA indicated a significant overall group effect, F(5,54) = 3.24. Subsequent post-hoc comparisons revealed that the group mean ABP responses of the naive and saline groups did not significantly differ, but these groups combined were significantly less than the group mean ABP obtained in the 24 hr group. Similar comparisons of the naive and saline control data with all other zymosan time conditions failed to attain statistical significance.

## Discussion

The most important finding of the present study was that the yeast cell wall component, zymosan, which evokes innate immune responses in a fashion similar to mycotic infections, led to an inflamed bladder and magnified reflex responses to bladder distension. Specifically, the present studies parametrically characterized multiple variables associated with the zymosan-induced model of bladder inflammation including the effect of time and solution concentration. Within the parameters tested, a single 30 minute instillation of a 1% zymosan solution resulted in a significant inflammatory response in the bladder as early as 4 hr after administration. This response peaked at 24 hr and by 48 hr was returning to control levels. This study also showed that both 1 and 2% concentrations produced significantly greater responses than the 0.1 and 0.5% concentrations. These findings are important to future studies related to this form of bladder inflammation.

It is notable that in the present studies there was an apparent temporal dissociation between local tissue inflammatory changes and the enhancement of reflex responses to bladder stimulation: statistically significant evidence of inflammation occurred prior to (4 and 12 hr) the development of robust and statistically significant enhancement of ABP and EMG responses to UBD which only occurred 24 hr after administration of zymosan. This peak timepoint for reflex enhancement was the peak time of inflammation as measured by Evan's Blue extravasation and so there was, at the very least, a rough correlation between the two endpoints. This observation is similar to those noted in studies using the stimulus of colorectal distension in which turpentine was used as an inflammatory agent [10]. In that study, there were early indications of inflammation but a robust enhancement of nociceptive responses required a longer period of time (24 hr) to become manifest. It is possible that the underlying physiological changes produced by zymosan-induced inflammation requires time for expression; perhaps due to delays related to axonal transport from the periphery to the central nervous system of an inflammogen-induced signal. Alternatively, it may be the case that the nociceptive measures are less sensitive than the employed measures of inflammation.

The neurophysiological basis for the presently observed increase in sensitivity of the bladder due to zymosan pretreatment is, as yet, unknown although both central and peripheral mechanisms of sensitization associated with bladder inflammation have been proposed [20]. Leading theories include the recruitment of previously "silent" nociceptors by pathological processes such as inflammation [21] or the sensitization of spinal dorsal horn neurons by the release of neuropeptides or glutamate [22]. These processes are not mutually exclusive and each may have variable relevance to acute versus chronic visceral pain disorders. A combination of both peripheral and central phenomena is likely in most acute pathological disorders whereby the activation of a specific subset of afferent neurons may lead to alterations in central nervous system processing. This has been demonstrated in models of somatic inflammatory pain and extrapolations to visceral models have been proposed [20]. The present study provides important parametric analyses related to zymosan-induced inflammation and secondary reflex changes that may allow investigators to use this form of inflammatory change in correlative neurophysiological studies.

The relation of inflammatory changes induced by topically applied agents to chronic pain disorders is also yet to be determined. Subjects with the painful bladder disorder interstitial cystitis typically describe their painful sensations as being similar to a urinary tract infection with its associated inflammation-induced hypersensitivity. The similarity of sensory reports from interstitial cystitis and urinary tract infections implies a similarity in neurophysiological changes along the neural pathway associated with bladder sensation. The precise level at which this convergence occurs can only be defined with a precise definition of the pathways involved with at least one of the sensations. Psychophysical studies of subjects with interstitial cystitis recently reported by ourselves [23] demonstrated subjects with interstitial cystitis to be hypersensitive to bladder distension as well as other to other deep tissue stimuli. This suggests altered central processing of sensory data, a phenomenon known to occur following inflammation in somatic systems which is generally described in terms of hyperalgesia (magnified responses to painful stimuli) or allodynia (pain evoked by normally non-painful stimuli). In the present study, reflex responses to UBD were magnified by zymosan application at intensities of 20 mm Hg or greater. Others [e.g., [20]] have proposed that UBD becomes "noxious" to rats when distension pressures exceed 40 mm Hg. This would suggest that the augmentation of responses to UBD produced by zymosan are at the very least representative of a visceral hyperalgesia-related phenomena and may even be related to phenomena such as allodynia. The difficulties of interpretation inherent to cross-species comparisons of subjective sensations limits further speculation.

## Conclusion

The present study parametrically examined responses to the intravesical administration of zymosan and demonstrated that this substance inflamed the bladder within 4 hr of administration and produced maximal inflammation 24 hr after topical administration. Statistically significant increases in ABP and EMG responses to UBD also occurred 24 hr post-zymosan administration but not at earlier timepoints. This suggests that any subsequent studies utilizing a single dose of intravesical zymosan to inflame the bladder would be optimized by a protocol in which pretreatment with a zymosan solution occurs 24 hr prior to subsequent measures.

## List of abbreviations

ABP – arterial blood pressure

EMG – electromyogram

UBD – urinary bladder distension

SEM – standard error of the mean

## Competing interests

The author(s) declare that they have no competing interests.

## Authors' contributions

AR and TN were involved in the design and implementation of studies, data collection & analysis and manuscript generation. TU and RC performed data collection and analysis.

## Pre-publication history

The pre-publication history for this paper can be accessed here:



## References

[B1] Sobel JD, Vazquez JA (1999). Fungal infections of the urinary tract. World J Urol.

[B2] Miller SI, Ernst RK, Bader MW (2005). LPS, TLR4 and infectious disease diversity. Nature Rev Microbiol.

[B3] Roeder A, Kirschning CJ, Rupec RA, Schaller M, Weindl G, Corting HC (2004). Toll-like receptors as key mediators in innate antifungal immunity. Med Mycol.

[B4] Levitz SM (2004). Interactions of Toll-like receptors with fungi. Microbes and Infection.

[B5] Kunz S, Tegeder I, Coste O, Marian C, Pfenninger A, Corvey C, Karas M, Geisslinger G, Niedeberger E (2005). Comparative proteomic analysis of the rat spinal cord in inflammatory and neuropathic pain models. Neurosci Letts.

[B6] Randich A, Meller ST, Gebhart GF (1997). Responses of primary afferents and spinal dorsal horn neurons to thermal and mechanical stimuli before and during zymosan-induced inflammation of the rat hindpaw. Brain Res.

[B7] Coutinho S, Meller ST, Gebhart GF (1996). Intracolonic zymosan produces visceral hyperalgesia in the rat that is mediated by spinal NMDA and non-NMDA receptors. Brain Res.

[B8] Ness TJ (1999). Models of visceral nociception. Ins Lab Animal Res J.

[B9] Ide Y, Maehara Y, Tsukahara S, Kitihata L, Collins G (1997). The effects of an intrathecal NMDA antagonist (AP5) on the behavioral changes induced by colorectal inflammation with turpentine in rats. Life Sci.

[B10] Ness TJ, Gebhart GF (2001). Inflammation enhances reflex and spinal neuron reponses to noxious visceral stimulation in rats. Am J Physiol Gastrointest Liver Physiol.

[B11] Burton M, Gebhart GF (1995). Effects of intracolonic acetic acid on responses to colorectal distension in the rat. Brain Res.

[B12] Langlois A, Diop L, Riviere P, Pascaud X, Junien J (1994). Effect of fedotozine on the cardiovascular pain reflex induced by distension of the irritated colon in the anesthetized rat. Eur J Pharmacol.

[B13] Julia V, Mezzasalma T, Bueno L (1995). Influence of bradykinin in gastrointestinal disorders and visceral pain induced by acute or chronic inflammation in rats. Dig Dis Sc.

[B14] Castroman P, Ness TJ (2001). Vigor of visceromotor response to urinary bladder distension in rats increases with repeated trials and stimulus intensity. Neurosci Lett.

[B15] Ness TJ, Lewis-Sides A, Castroman P (2001). Characterization of pressor and visceromotor reflex responses to bladder distension in rats: sources of variability and effect of analgesics. J Urol.

[B16] McMahon S, Abel C (1987). A model for the study of visceral pain states: chronic inflammation of the chronic decerebrate rat urinary bladder by irritant chemicals. Pain.

[B17] Koltzenburg M, McMahon S (1986). Plasma extravasation in the rat urinary bladder following mechanical, electrical and chemical stimuli: evidence for a new population of chemosensitive primary sensory afferents. Neurosci Lttrs.

[B18] Saria A, Lundberg J (1983). Evans blue fluorescence: quantitative and morphological evaluation of vascular perimeability in animal tissues. J Neuroci Methods.

[B19] Anderson R, Ness T, Gebhart G (1988). A distension control device useful for quantitative studies of hollow organ sensation. Physiol Behav.

[B20] McMahon SB, Dmitrieva N, Koltzenberg M (1995). Visceral pain. Br J Anaesth.

[B21] Michaelis M, Habler HJ, Jaenig W (1996). Silent afferents: a separate class of primary afferents?. Clin Exp Pharmacol Physiol.

[B22] Ishigooka M, Zermann DH, Doggeweiler R, Schmidt RA, Hashimoto T, Nakada T (2001). Spinal NK1 receptor is upregulated after chronic bladder irritation. Pain.

[B23] Ness TJ, Powell-Boone T, Cannon R, Lloyd LK, Fillingim RB (2005). Psychophysical evidence of hypersensitivity in subjects with interstitial cystitis. J Urol.

